# Classification and Segmentation of Longitudinal Road Marking Using Convolutional Neural Networks for Dynamic Retroreflection Estimation

**DOI:** 10.3390/s20195560

**Published:** 2020-09-28

**Authors:** Chanjun Chun, Taehee Lee, Sungil Kwon, Seung-Ki Ryu

**Affiliations:** 1 Future Infrastructure Research Center, Korea Institute of Civil Engineering and Building Technology (KICT), Goyang 10223, Korea; chanjunchun@kict.re.kr (C.C.); thlee420@kict.re.kr (T.L.); 2 HI-LANDKOREA Co., Ltd., Seoul 05045, Korea; hilandkorea@naver.com

**Keywords:** retroreflection, longitudinal road marking, luminance, convolutional neural network, classification, segmentation

## Abstract

Road markings constitute one of the most important elements of the road. Moreover, they are managed according to specific standards, including a criterion for a luminous contrast, which can be referred to as retroreflection. Retroreflection can be used to measure the reflection properties of road markings or other road facilities. It is essential to manage retroreflection in order to improve road safety and sustainability. In this study, we propose a dynamic retroreflection estimation method for longitudinal road markings, which employs a luminance camera and convolutional neural networks (CNNs). The images that were captured by a luminance camera were input into a classification and regression CNN model in order to determine whether the longitudinal road marking was accurately acquired. A segmentation model was also developed and implemented in order to accurately present the longitudinal road marking and reference plate if a longitudinal road marking was determined to exist in the captured image. The retroreflection was dynamically measured as a driver drove along an actual road; consequently, the effectiveness of the proposed method was demonstrated.

## 1. Introduction

Road markings constitute one of the most important elements of a road. During the daytime, drivers perceive road markings because of the visual contrast between the road surface and road marking. However, the luminous contrast becomes more important when the visual conditions are poor at night and during bad weather. These road markings are managed according to specific standards, which may slightly differ, depending on the country [1]. Among the various types of road markings, longitudinal road markings can be considered to be particularly important, and it is common to have relatively stricter standards for them. These standard criteria include a criterion for a luminous contrast, which can be referred to as retroreflection [2,3]. On a clear day, the absolute luminance is increased; conversely, on a rainy and overcast day, the absolute luminance is relatively low. Thus, instead of measuring the absolute luminance, it is better to measure the retroreflectivity according to the angle at which the vehicle lights illuminate the road [4].

Static and dynamic methods are used in order to measure retroreflection [4]. In the case of static retroreflection measurement, a person measures road markings one-by-one from the outside. This static measurement equipment is relatively inexpensive when compared to the dynamic equipment, but it takes a relatively large amount of time and cost to measure a large amount of roads. Furthermore, it is possible to cause traffic congestion, thereby increasing the traffic risk. In the case of the dynamic measurement equipment, it is possible to measure retroreflection during actual driving by attaching it to the outside of the vehicle. Therefore, it is relatively comfortable to inspect a large amount of roads when compared to static, but it is comparatively very expensive.

**Figure 1:**
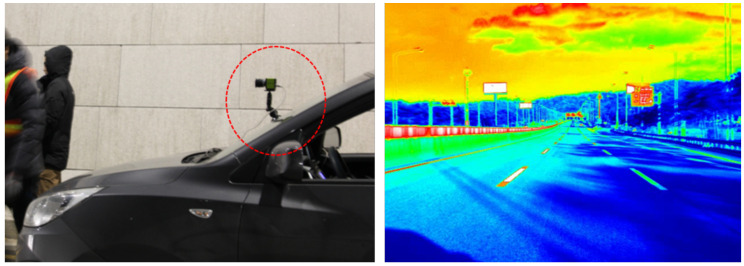
Example of a luminance camera setup and captured image.

Figure 1 shows a luminance camera used in this study, and an example captured image. Note that the captured image in the figure is only presented in color for visual clarity, as the data were recorded as one-channel pixel-by-pixel luminance values. This equipment was used in order to measure the dynamic luminance; the cost was similar to that of static retroreflection measurement equipment. This type of luminance camera measures the absolute luminance; the luminance values may considerably differ, depending on the weather and time of day, even when the camera is applied to the same road marking. However, the application of a well-managed reference may be used to overcome this problem. More specifically, a reference can be used to compare the luminance values of a single road marking under different visibility conditions. In this paper, this reference is referred to as the reference plate. For dynamic measurements, the specific position of the reference plate and road marking in the captured image must be known to extract the luminance values. Moreover, because the vehicle is in motion during these measurements, at least one road marking will be present in each captured image. Thus, the measurement system must be able to determine whether a road marking exists.

Image-processing technologies have significantly progressed in recent years, owing to the emergence of deep neural networks. In particular, convolutional neural network (CNN)-based systems are popular among the various types of neural networks [5]. CNN-based algorithms are increasing in popularity among competitors in the ImageNet Large Scale Visual Recognition Competition (ILSVRC), which evaluates algorithms that are based on their ability to overcome classification and detection problems [6,7]. CNN-based algorithms have also been demonstrated to perform better than conventional image-processing algorithms when it comes to addressing regression problems [8], object detection [9], and semantic segmentation [10,11].

In this paper, we propose a CNN-based dynamic pseudo-retroreflection estimation method for longitudinal road markings, which entails the use of a luminance camera. To begin, the luminance camera captures an image that provides input data to a classification and regression CNN model, which is purposed to determine whether a longitudinal road marking exists in each captured image. Here, conventional CNN models that demonstrated good performance in the ImageNet competition were employed as backbones. If a longitudinal road marking is determined to exist in the captured image, then a segmentation model that appropriately segments the longitudinal road marking and reference plate is implemented. When implemented in sequence, these two models extract the average luminance values of the longitudinal road marking and reference plate, and the retroreflection is calculated while using the average luminance values. In this study, dynamic retroreflection measurements were performed as an operator drove along an actual road. As such, only the dynamic retroreflection data for longitudinal road markings were automatically acquired.

This paper is organized, as follows. Following the Introduction, Section 2 introduces the proposed framework for dynamic retroreflection measurement. Section 3 describes the classification and regression model that was used to determine whether the longitudinal road markings were accurately acquired in the luminance image. Section 4 introduces the segmentation model, which was used to precisely delineate the area of each longitudinal road marking with respect to the reference plate. In Section 5, the results of the dynamic retroreflection measurements are presented and discussed. Lastly, Section 6 concludes this study.

## 2. Proposed Framework for Dynamic Retroreflection Estimation

**Figure 2:**
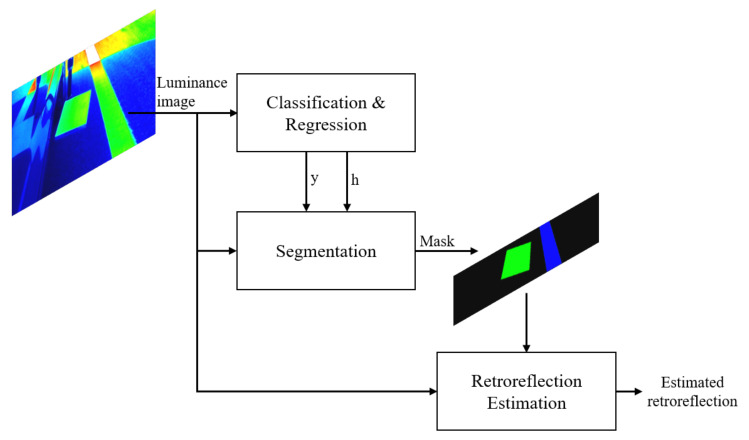
Proposed framework for dynamic retroreflection estimation.

Figure 2 shows the proposed framework for dynamic retroreflection estimation. The proposed framework receives luminance image data that were acquired by a luminance camera. Here, the luminance image is expressed as a color image, but it is actually one-channel image with a pixel-by-pixel luminance value. First, the neural network determines whether the luminance image data can be used to make a retroreflection prediction. If a prediction is possible, then the luminance image is cropped to identify the retroreflection. Subsequently, the reference plate and longitudinal road marking are precisely segmented. Thus, the proposed neural network can be divided into a model that determines whether retroreflection can be extracted, and a model that performs segmentation. The absolute luminance value can significantly vary, depending on various factors, such as the weather conditions. However, with the proposed system, we can predict pseudo-retroreflection by comparing the luminance values of the reference plate and longitudinal road markings.

**Figure 3:**
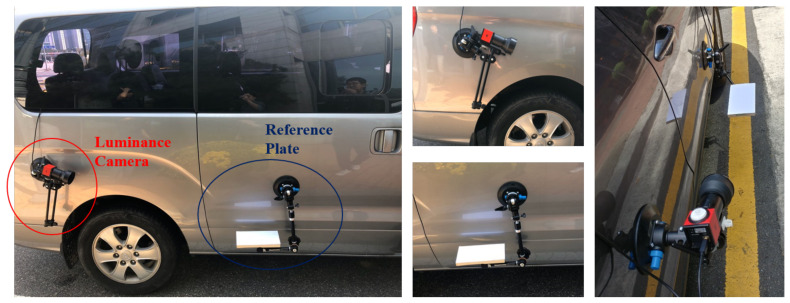
Placement of the luminance camera and reference plate on a vehicle used to capture luminance images.

**Figure 4:**
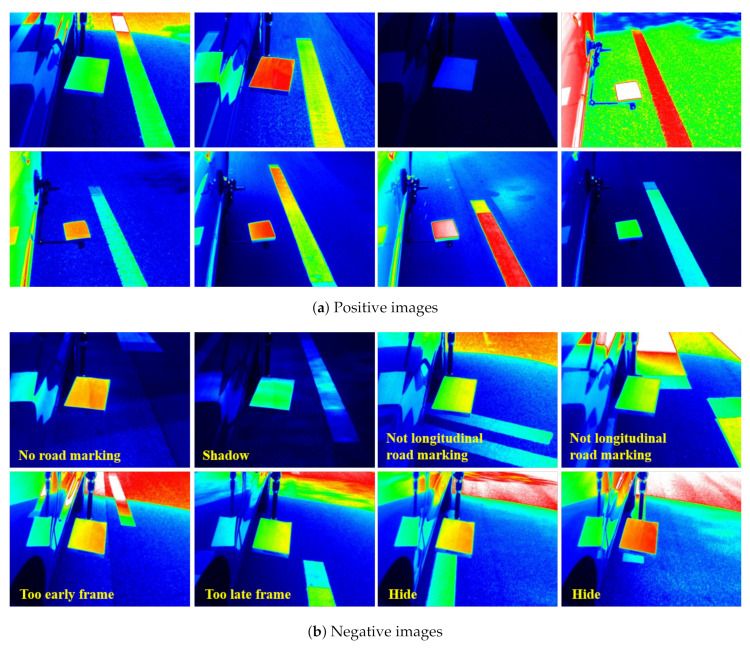
Examples of positive and negative images.

We first constructed a luminance dataset to train these two models. Figure 3 shows the placement of the luminance camera and reference plate that were installed on a vehicle to acquire luminance images. The luminance camera installed at the rear was placed to ensure simultaneous capture of the reference plate and longitudinal road marking. This luminance camera has a 6 mm fixed focus lens, and it can measure luminance in the range of 10–100,000 cd/m^2^. In addition, the obtained luminance image has a resolution of 640 × 480 pixels. The image data were acquired as an operator drove along an actual road at speeds that did not exceed 70 km/h. The maximum speed complies with the recommendations of the manufacturer of the luminance camera. Dynamic luminance image data were obtained over a period of seven days, with each driving session lasting for an average of five hours. A total of 29,635 luminance images were captured (Table 1). A total of 24,270 images were used for training and 5365 images were employed for evaluation. In this table, “positive” and “negative” indicate whether the reference plate and longitudinal road marking image data could be used in order to accurately determine the luminance. Note that the number of positive images is only 20% of the number of negative images. It tends to be much more imbalanced in the process of acquiring luminance images, but we tried to supplement the positive images. We designated a driving route that can extract appropriately positive images. As can be ascertained from the table, various factors prevented accurate luminance value extraction. Figure 4 shows examples of positive and negative images. In the positive images, the reference plate and longitudinal road marking were properly captured. Conversely, in the negative image examples, the reference plate and longitudinal road marking could not be properly segmented. The reasons for this are as follows:

- There was no longitudinal road marking in the image.
- Road markings other than longitudinal road markings were captured.
- The luminance could not be accurately measured, because only the reference plate or longitudinal road marking had a shadow.
- The longitudinal road marking was not in a position similar to that of the reference plate, implying that it was captured too early or too late.
- The reference plate or vehicle was obstructing the longitudinal road marking; thus, it was not clearly visible.

For these reasons, positive and negative images were distinguished while using human labels. Although we attempted to maintain a consistent standard, numerous images had very ambiguous boundaries. Thus, the image dataset was constructed to exclude these images.

**Figure 5:**
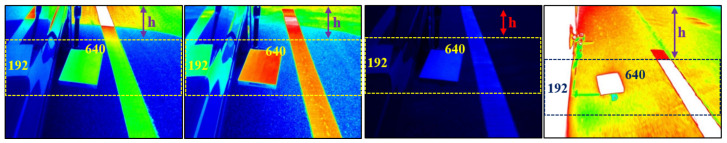
Examples of cropped positive images that captured the longitudinal road marking in close proximity to the reference plate.

If the image is taken as “positive”, then *h* is also predicted. Here, *h* refers to the height that is denoted in Figure 5, the images of which have been cropped to only include the longitudinal road marking near the reference plate. The reliability of the luminance value was assumed to be dependent on the position of the longitudinal road marking with respect to the reference plate. Note that finding the correct *h* can be ambiguous. Here, it was judged that h was well annotated if the reference plate was located in the middle of the cropped image. The cropped images were fed to the neural network model performing the segmentation task. The classification, regression model, and segmentation model are described in detail in the following sections.

## 3. Classification and Regression Model

### 3.1. Neural Network

**Figure 6:**
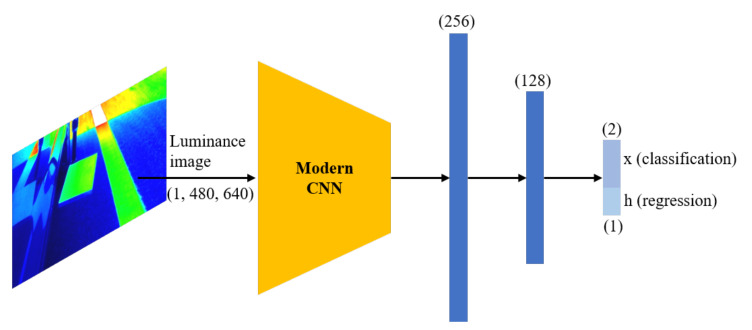
Neural network architecture of convolutional neural network (CNN) model for classification and regression. Pretrained models, i.e., Resnet-18 [12], VGG-16 [13], Alexnet [6], Mobilenet [14], and Shufflenet [15], were employed as backbones.

Figure 6 shows the neural network architecture for classification and regression. In the CNN layer, pretrained models from ImageNet [7] were utilized. ImageNet has a very large image database that is organized according to the WordNet hierarchy. The year 2017 marked the last year for the ImageNet challenge; at this time, the classification task was to classify 1000 classes [7]. CNN-based models have demonstrated good performance since 2012, and, thus, have been employed for various image classification tasks by utilizing the pretrained models made available via the ImageNet competition. The CNN models utilized as a backbone in this study are, as follows:

- Resnet-18 [12]
- VGG-16 [13]
- Alexnet [6]
- Mobilenet [14]
- Shufflenet [15]

These CNN models were selected, because they demonstrated very good performance on ImageNet challenges. The original models have 1000 classes, but, in this study, only binary classification exists. Furthermore, there is also an output that predicts the value of *h* to optimize the image cropping process. Therefore, the last layers were modified such that a hidden layer with 256 and 128 units was added, and only three units were estimated at the output layer. Note that the same fully connected layer with different objective functions is used for regression and classification. It was equally applied to all models; the code is available at https://github.com/cjchun313/Retroreflection_Estimation.

The numbers of training and evaluation images were 24,270 and 5365, respectively, as described in Table 1. Note that we did not apply any image augmentation to fix the angle of the road marking and the luminance value. Regarding positive and negative image classification, cross entropy was employed as a loss function, and the mean squared error was utilized to predict the *h* value [5]. Adaptive moment estimation (Adam) was used as the optimization technique [16]. Adam stores the exponentially decaying averages of past gradients and squared gradients, such that

(1) $$\theta_{t+1}=\theta_{t}-\frac{\eta }{\sqrt{v_{t}+\epsilon }}\frac{\sqrt{1-\beta_{2}^{t}}}{1-\beta_{1}^{t}}m_{t}$$

where

(2) $$\begin{array}{c} m_{t}=\beta_{1}m_{t-1}+\left(1-\beta_{1}\right)g_{t}, \end{array}$$

(3) $$\begin{array}{c} v_{t}=\beta_{2}v_{t-1}+\left(1-\beta_{2}\right)g_{t}^{2}. \end{array}$$

Here, $\beta_{1}$ and $\beta_{2}$ were set to 0.9 and 0.999, respectively. All of the models had a total of 50 epochs, the initial learning rate was set to equal 0.001, and a decaying factor of 0.8 was applied every 10 epochs. The ReLU function was used as the activation function [17], such that f(x)=max(0,x). In this study, we used Quadro RTX 8000 with 48 GB memory for training, and it took approximately two hours per model.

### 3.2. Performance Evaluation

**Figure 7:**
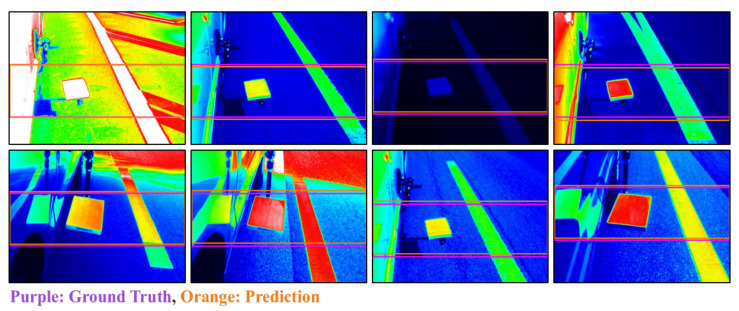
Comparative analysis for the Resnet-18 model *h* prediction result and ground truth.

The accuracy of classification and mean-absolute error (MAE) of the regression were calculated in order to compare the effectiveness of the trained models. A total of 1025 positive and 4340 negative images were utilized for this evaluation. Table 2 presents the classification and regression performance results for each model. Regarding classification accuracy, the Resnet-18 and AlexNet models were found to have the best and worst performance, at 96.9618 and 95.0792, respectively. Regarding the regression performance, Mobilenet demonstrated the best performance, achieving an MAE value as low as 0.0169; as was observed with the classification performance, AlexNet performed the worst (MAE = 0.1127). Because the *h* value ranged from 0 to 480, an MAE value of 0.0169 can be interpreted as an average error of approximately eight pixels. In the case of AlexNet, the MAE value of 0.1127 implies an average error of approximately 54 pixels, which corresponds to an error that is slightly above 10%. Figure 7 presents examples of the discrepancy between the *h* prediction result and ground truth for the Resnet-18 model. All of the prediction results include exactly the reference plate, which was found to be considerably similar to the ground truth, as can be seen in the figure.

## 4. Segmentation Model

### 4.1. Neural Network

The reference plate and longitudinal road markings were segmented in each cropped image output by the classification and regression model described in the previous section. In the proposed model, if the areas of the reference plate and longitudinal road marking are precisely segmented, then the average luminance of the reference plate and longitudinal road marking can be calculated. Subsequently, the pseudo-retroreflection can be estimated while using these average luminance values.

**Figure 8:**
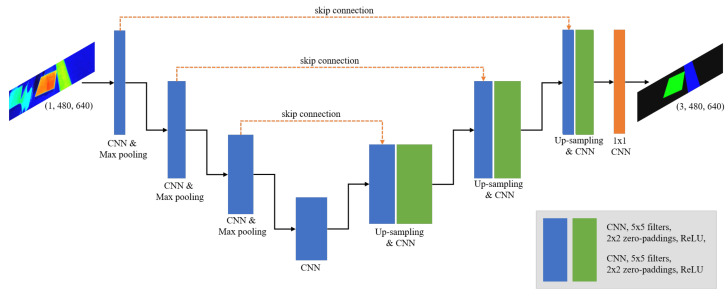
Architecture of segmentation CNN model.

Figure 8 shows the neural network architecture applied for segmentation. We utilized U-Net, which is widely employed for segmentation tasks, as the basic framework for our segmentation model [18]. Although it was developed for biomedical image segmentation, it is also applied in the road traffic domain [19]. Additionally, U-Net is similar to a fully convolutional autoencoder [11]. A characteristic of this type of autoencoder is that it temporarily reduces the size of the input image, i.e., the image size is reduced and subsequently increased in order to match that of the original input image. Thus, because the procedure includes processes that mimic encoding and decoding processes, this type of neural network has been termed an autoencoder [20,21]. The autoencoder is employed in unsupervised learning, but this neural network architecture for semantic segmentation is employed in order to perform pixel-by-pixel supervised classification [10].

Note that the number of training and evaluation images were 3933 and 1025, respectively. Only positive images were employed for segmentation. Accordingly, cross entropy was employed as a loss function and Adam was used as the optimization technique [16]. Note that all other hyperparameters were identical to those that were applied in training the classification and regression model.

### 4.2. Performance Evaluation

**Figure 9:**
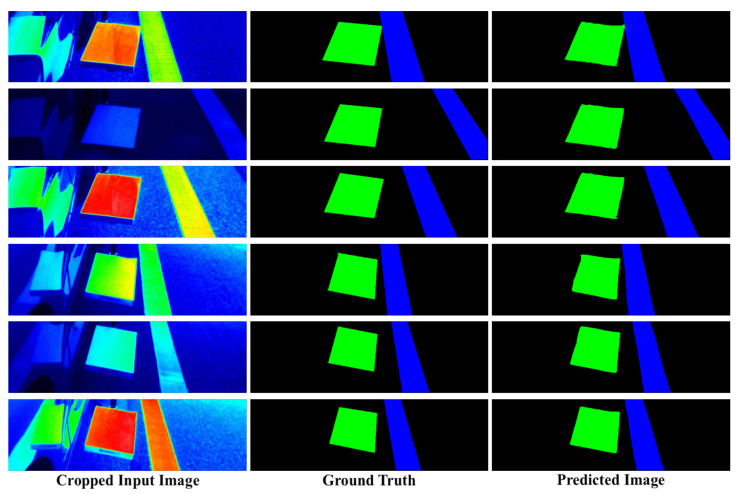
Examples of ground truth and segmented (predicted) results.

The segmentation model was also implemented in order to perform objective evaluation and verify the overall system performance. Note that, as previously mentioned, only 1025 images were input into this model, because negative images were not suitable for segmentation. Additionally, the accuracy and MAE were measured in the classification and regression model, and the intersection-over-union (IoU) was measured in the segmentation model [22]. The IoU is a widely used metric to evaluate the segmentation or detection tasks. Table 3 presents the IoU measurement results for the segmentation model. The IoU values for the background, reference plate, and longitudinal road marking were 0.9860, 0.9349, and 0.9495, respectively, which corresponded to an average IoU value of 0.9568. High IoU values were obtained for the background, reference plate, and longitudinal road markings. The reference plate and longitudinal road markings have relatively uniform shapes as compared to other target objects for segmentation or detection tasks. It is for this reason that this segmentation model was able to achieve such high performance. Figure 9 provides examples of the ground truth and segmented (predicted) results. As can be seen in the figure, the predicted results were very similar to the ground truth.

## 5. Result and Discussion

**Figure 10:**
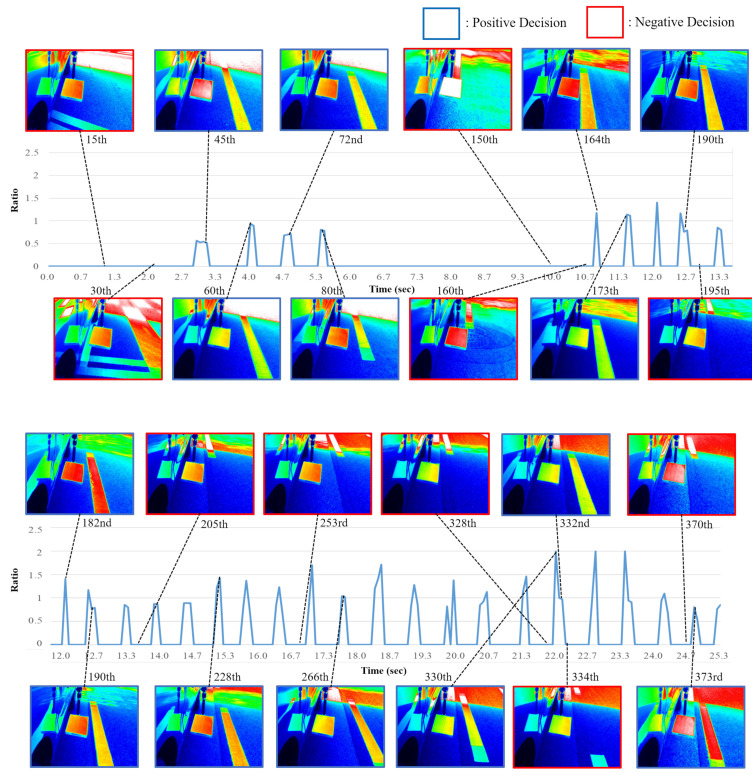
Predicted dynamic retroreflection results.

A predictive, CNN-based dynamic retroreflection method was developed, as described in Section 2. Figure 10 shows the results of dynamic retroreflection prediction. The horizontal axis denotes the time index and the vertical axis denotes the ratio, which provides information regarding the relationship between the average luminance of the longitudinal road marking and that of the reference plate. Note that, when the classification model yields a negative image, the ratio is 0. When the average luminance value for the longitudinal road marking is higher than that for the reference plate, the ratio exceeds 1. Additionally, in the proposed system, all of the predicted images with a ratio above 2 are subjected to clipping. It should also be noted that the blue- and red-framed captured luminance images correspond to positive and negative predictions, respectively. As described in the previous section, the classification accuracy was high and, as can be ascertained from the results presented in the figure, segmentation was properly carried out. However, the proposed neural network structure has the following problems:

- When the classification model yields a false-positive result, the longitudinal road marking segmentation result is unreliable.
- The segmentation model perceives the images reflected onto the vehicle as the reference plate.
- The segmentation model perceives other road markings as a longitudinal road marking.

A false-positive classification result occurs when the classification model has determined that a longitudinal road marking exists in an image, although no longitudinal road marking is actually present. Under these conditions, the segmentation model has to segment the longitudinal road marking. In this case, the segmentation model yields incorrect parts. There were also instances, in which an image reflected onto the vehicle was very similar to the actual reference plate. In this case, the segmentation model tends to present the reflected image instead of the actual reference plate. Additionally, other road markings were sometimes mistaken for longitudinal road markings. This was more likely to occur when the vehicle made a left or right turn, rather than when it was proceeding along a straight path.

It should also be noted that we attempted to install the reference plate and luminance camera in the same respective positions on the vehicle. It is necessary to verify how these things influence retroreflection measurements. Moreover, it is also essential to verify the correlation between the predicted level of retroreflection that was estimated by the proposed method and the actual level of retroreflection.

## 6. Conclusions

A reference plate and luminance camera were installed on an actual vehicle in order to estimate the level of retroreflection of the longitudinal road markings. As an operator drove along roads, the luminance camera captured images. A total of 29,635 luminance images were captured; among them, 4958 and 24,677 were positive and negative images, respectively. The acquired image data were used to train novel CNN-based classification and regression models, which were constructed while using conventional CNN models as backbones; consequently, good classification and regression performance was confirmed. Furthermore, the segmentation results confirmed that the reference plate and longitudinal road markings were accurately presented. Additionally, the mask that was produced by the segmentation model was used to extract the average luminance values of the reference plate and longitudinal road markings, such that the relationship could be determined. We regarded this relationship as a dynamic pseudo-retroreflection predictor for longitudinal road markings, and assumed that it would be correlated with the actual level of retroreflection. In the future, it will be necessary to verify the correlation between the level of retroreflection estimated via the proposed method and the actual level of retroreflection.

## Figures and Tables

**Table 1:** Numbers of luminance images for training and evaluation.

	Train			Evaluation		
	Positive	Negative	Total	Positive	Negative	Total
Number of images	3933	20,337	24,270	1025	4340	5365

**Table 2:** Comparison of the classification and regression performance of each model.

	Accuracy (%) (Classification)	MAE (Regression)
Resnet-18	96.9618	0.0230
VGG-16	96.5704	0.0631
Alexnet	95.0792	0.1127
Mobilenet	96.7568	0.0169
Shufflenet	96.8872	0.0305

**Table 3:** Intersection-over-union (IoU) results obtained via the segmentation model.

	Background	Reference Plate	Longitudinal Road Marking	Average
IoU	0.9860	0.9349	0.9495	0.9568
